# Clinicopathological and ultrastructural characterization of periapical actinomycosis

**DOI:** 10.4317/medoral.23247

**Published:** 2019-12-24

**Authors:** Wagner Gomes-Silva, Débora Lima Pereira, Eduardo Rodrigues Fregnani, Oslei Paes Almeida, Luciana Armada, Fábio Ramôa Pires

**Affiliations:** 1 Departamento de Diagnóstico Oral, Faculdade de Odontologia de Piracicaba, Universidade Estadual de Campinas, Piracicaba, São Paulo, Brasil; 2 Departamento de Saúde III, Faculdade de Ciências Médicas, Universidade Nove de Julho, São Paulo, São Paulo, Brasil; 3 Departamento de Medicina Oral, Hospital Sírio-Libanês, São Paulo, São Paulo, Brasil; 4 Programa de pós-graduação em Odontologia, Universidade Estácio de Sá, Rio de Janeiro, Rio de Janeiro, Brasil; 5 Departamento de Diagnóstico e Terapêutica, Faculdade de Odontologia, Universidade do Estado do Rio de Janeiro, Rio de Janeiro, Rio de Janeiro, Brasil

## Abstract

**Background:**

The aim of the present study was to analyze the clinicopathological and the ultrastructural features of periapical actinomycosis (PA) cases.

**Material and Methods:**

Data from the files of an oral pathology laboratory were retrieved and the findings of histopathological analysis were evaluated. Hematoxylin–eosin (HE), a modified Brown & Brenn, and Grocott stains as well as ultrastructural analysis using scanning electron microscopy (SEM) and energy dispersive X-ray spectroscopy (EDX) were utilized.

**Results:**

Six cases were obtained, 4 females and 2 males, with a mean age of 34 year-old. Two cases were symptomatic, lower teeth and the anterior region were more commonly affected, and all cases were characterized by periapical radiolucencies. All cases presented sulfur granules with a ray-fungus or club-shaped pattern of the Splendore-Hoeppli phenomenon in HE-stained sections, with filamentous gram-positive bacteria aggregates highlighted by the modified Brown & Brenn stain. SEM analysis revealed abundant packed rod-like and filamentous bacteria associated with an extracellular amorphous material. EDX analysis showed predominant picks of calcium and sulfur in actinomycotic colonies.

**Conclusions:**

Our findings suggest that PA manifests either clinically and radiologically as a non-specific and heterogeneous condition and that the actinomycotic colonies consist in a calcium- and sulfur-rich matrix. Furthermore, the results highlight the importance of submitting periapical specimens after surgical removal to histopathological analysis.

** Key words:**Actinomyces, actinomycosis, periapical diseases.

## Introduction

Actinomycosis is a chronic infectious disease caused by obligatory or facultative anaerobic gram-positive bacteria belonging to the genus Actinomyces (1,2). It was firstly described in humans probably in 1878 by Israel and Wolfe, who isolated these organisms in culture (3,4). The term Actinomyces was derived from the morphological appearance of these microorganisms that resembled fungal hyphae and were unveiled bacillary filamentous aggregates later (5).

Actinomycosis is an uncommon infection characterized by a wide spectrum of clinical presentations, including abscess formation, fistulas and fibrosis, with potential to soft and hard tissues involvement in a variable course (1,6). At least four different clinical forms of actinomycotic human infections (cervicofacial, pulmonary/thoracic, abdominopelvic and cerebral) (6,7) have been well-documented, although other forms such as periapical actinomycosis (PA) have been also described (8,9). PA usually reveals to be an indolent, chronic and local infection indistinguishable clinically and radiologically from conventional apical periodontitis (9). Therefore, the final diagnosis is usually achieved only after surgical removal of the lesion and histopathological examination of the specimen.

In the present study, we characterized the clinicopathological and ultrastructural features of six PA cases, emphasizing the importance of submitting periapical specimens after surgical removal to histopathological analysis.

## Material and Methods

A retrospective review was performed at the files of one oral pathology laboratory and all cases of PA were selected. Clinical and radiological information were retrieved from the laboratory records. Histological description and diagnosis confirmation of each case were performed in 5-µm sections on hematoxylin and eosin (HE)-stained slides. Additional sections were subjected to a modified Brown & Brenn (10) and Grocott stains to confirm the presence of filamentous gram-positive Actinomyces in the tissues. This study was approved by the local ethics committee (Hospital Universitário Pedro Ernesto/UERJ) under the protocol number 536.544 and was conducted in accordance with the Declaration of Helsinki to human studies, including informed consent form application.

In addition, ultrastructural analyses were performed using scanning electron microscopy (SEM; JEOL JSM-5600LV) for characterization of the actinomycotic colonies and energy dispersive X-ray spectroscopy (EDX; Vantage system, Noran Instruments, Software EasyMicro) for analysis of the chemical content. Inflamed connective tissue from the same PA histological section was used as internal control in EDX analysis.

## Results

**Figure 1:**
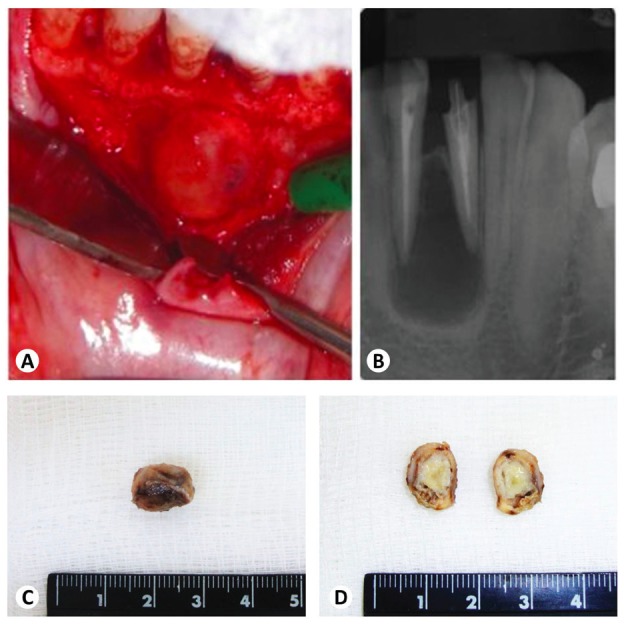
Clinical and gross analysis. (A) Intraoperative view of case 4, showing an intraosseous cystic lesion emerging between the right lower central and lateral incisors roots. (B) Preoperative periapical radiograph showing a well-defined radiolucent lesion involving both periapical regions, which presented well-performed endodontic treatment. (C) Gross image of the surgically-removed specimen from the same case, showing a brownish and ovoid mass. (D) It can be observed a yellowish sulfur granule filling the whole cystic cavity of the lesion after the gross section.

Six cases of PA were enrolled in this study and all six patients with PA underwent surgical curettage/enucleation of the lesions as part of the treatment. Four patients were females and 2 males, with a mean age of 34 year-old, ranging from 19 to 65 year-old. Five cases affected the anterior region (3 in the mandible and 2 in the maxilla), particularly, the mandibular central incisors area. One additional case affected a first mandibular molar. In three cases, endodontic treatment was considered well-performed. One case was located in an edentulous area with no association with permanent teeth, rendering an image compatible with a residual cyst. Two patients were symptomatic and complained of swelling with purulent discharge in the affected area and pain, respectively. A yellowish appearance of the lesion was reported and could be confirmed in the intraoperative view in another case (Fig. 1).

The mean radiological size of the lesions was 1.6 cm, ranging from 1.0 to 2.0 cm. All lesions were radiolucent and 3 presented well-defined borders (Fig. 1). External radicular resorption was not reported in any of the cases. Clinical and radiological differential diagnosis included mostly periapical cysts, but also included odontogenic abscesses and other odontogenic cystic lesions. Table 1 presents a summary of the available clinical and radiological information from the 6 cases.

Gross analysis of the surgical specimens revealed fibroelastic soft tissue fragments that were predominantly brown in color and varied in shape from round/ovoid to irregular (Fig. 1). The mean volume of the specimens was 1.8 cm3 (0.27-3.63 cm3). Macroscopic structures compatible with sulfur granules were observed in three specimens presenting a cystic appearance (Fig. 1) but cystic lining-epithelium was encountered in only 2 cases. Gross and histopathological information are summarized in Table 2.

**Figure 2:**
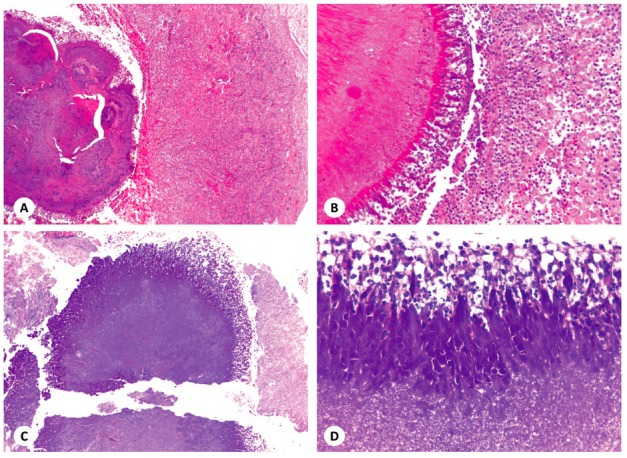
Histopathological analysis. (A) Actinomycotic sulfur granule, on the left, surrounded by an intense infiltrate of inflammatory cells (HE; original magnification, ×50). (B) In detail, the periphery showing the ray-fungus appearance with the Splendore-Hoeppli phenomenon and mainly neutrophils as well as some plasma cells, macrophages and lymphocytes in close contact with the bacterial colony (HE; original magnification, ×100). (C) A periapical granuloma displaying a central actinomycotic colony with the Splendore-Hoeppli phenomenon (HE; original magnification, ×50). (D) In a high power view, it is possible to see the radiating club-pattern at the periphery of the sulfur granule with intermixed and surrounding immune cells (HE; original magnification, ×400).

Under optical light microscopy, all cases presented detecTablesulfur granules showing a ray-fungus or club-shaped pattern of Splendore-Hoeppli phenomenon, consisting in actinomycotic colonies composed of masses of amorphous material with a radiating eosinophilic peripheral pattern surrounded by an inflammatory infiltrate composed mainly of neutrophils, as well as lymphocytes, plasma cells and macrophages (Fig. 2).

None of the cases presented isolated actinomycotic colonies without inflammatory cells that could be misinterpreted as floated bacterial colonies contamination.

**Figure 3:**
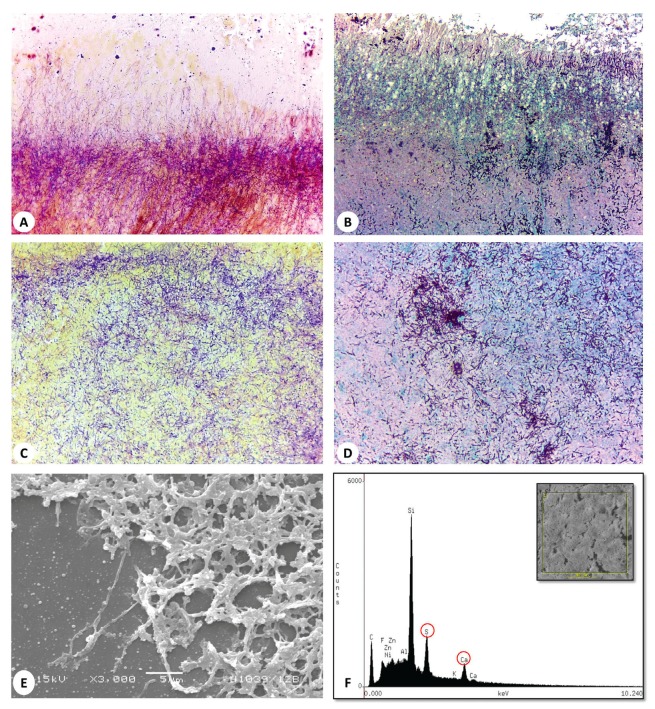
Histochemical staining and ultrastructural analysis. (A) Brown & Brenn’s stain showing dense aggregates of filamentous actinomyces in the periphery (Taylor’s modified Brown & Brenn; original magnification, ×200). (B) Grocott’s stain showing argyrophilic filaments compatible with Actinomyces at the periphery of a sulfur granule (Grocott stain; original magnification, ×200). (C) The central area of a sulfur granule with diffuse bacterial aggregates (Taylor’s modified Brown & Brenn; original magnification, ×200). (D) A central area showing filamentous bacteria positive to Grocott stain (Grocott stain; original magnification, ×200). (E) Scanning electron micrograph showing packed rod-like and filamentous bacteria with interspersed coccoid and other bacterial types (original magnification, ×3000). (F) Energy dispersive X-ray spectroscopy graphic representing the analysis of the correspondent sulfur granule area (box in the upper right corner) showing picks of sulfur and calcium. The area shows characteristic filamentous Actinomyces associated with an amorphous extracellular matrix and nucleus of calcification.

Modified Brown & Brenn stain revealed filamentous gram-positive bacteria dispersed in the sulfur granules and dense aggregates concentrated in the peripheral and central areas (Fig. 3). Other types of gram-positive bacteria were also admixed in the granules in fewer amounts. Grocott staining showed variable positivity, with some specimens demonstrating argyrophilic filaments and granules that were consistent with filamentous Actinomyces (Fig. 3).

SEM analysis revealed abundant packed rod-like and filamentous bacteria associated with an extracellular amorphous material and calcification. Coccoid microorganisms were also seen (Fig. 3). EDX analysis showed predominant picks of sulfur and calcium apart from silica and carbon that actually represented part of glass slide preparation (Fig. 3). The actinomycotic areas differed substantially when compared to the inflamed connective tissues used as internal controls.

**Table 1:**
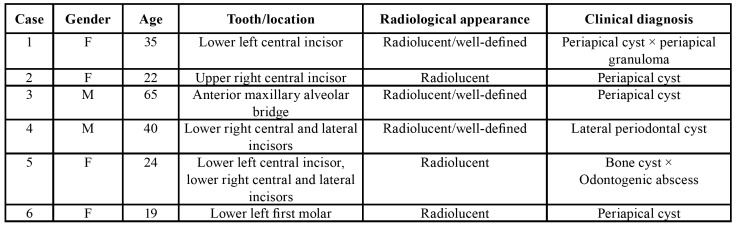
Clinical and radiological features from the 6 cases of periapical actinomycosis.

**Table 2:**
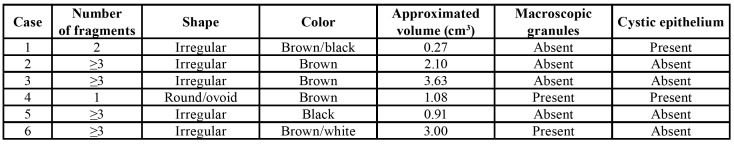
Gross and histopathological features of the 6 cases of periapical actinomycosis.

## Discussion

Although Actinomyces can live commensally in human cavities such as the mouth, oropharynx, gastrointestinal and female genital tracts, they can occasionally cause different forms of infection (11). Disruption of the integrity of the mucosa, which is the primary physical barrier, is considered important to the onset of actinomycotic infections (12). However, the underlying pathogenic mechanisms for this infection remain unclear, and, depending on the affected site, the infection can show heterogeneous behavior and different clinical presentations (6,7).

The most common type of actinomycosis is the cervicofacial form which accounts for approximately 50% of all cases and shows a male predominance of 1.5-3.1 times (11,13). Dental procedures such as dental extractions and oral conditions such as caries, periodontal disease and poor oral hygiene seem to play a critical role in its occurrence (11,13). Additionally, factors such as diabetes, malnutrition, immunosuppression and head and neck radiotherapy have been also considered to significantly increase the risks for this form of actinomycosis (13). It presents typically as a chronic, slow and non-tender lesion that progress to multiple abscesses, fistulae, and draining sinus tracts.

Unlike the cervicofacial form, PA is considered an uncommon, more indolent and restricted infection that has been reported in less than 5% of periapical specimens submitted for oral pathology laboratories (9,14-16). Nonetheless, it can be substantially underreported, considering the number of periapical surgical specimens that are not submitted to histopathological analysis after removal, as well as the differences on reporting these lesions by different oral pathology laboratories. Using previous published data of the same oral pathology laboratory (17), prevalence of PA could be estimated in less than 1% of all periapical lesions submitted to histological examination in our series.

More likely, authors have characterized PA when sulfur granules with the Splendore-Hoeppli phenomenon are present, which consists in eosinophilic material around filamentous gram-positive colony-forming aggregates surrounded by inflammatory cells, probably composed of antigen-antibody complex, tissue debris and fibrin. Notwithstanding, a recent study detected Actinomyces israelii’s DNA in radicular cysts that did not present the actinomycotic colonies in histological sections (18). Whether Actinomyces DNA detection in periapical lesions can be secondary or a determinant factor for the infection is still unclear. Moreover, the presence of sulfur granules with the Splendore-Hoeppli phenomenon as a mandatory requirement to establish the diagnosis of PA is controversial and unspecific since other conditions can share this feature. There has been no consensus in PA diagnostic criteria, even though all the six cases demonstrated this feature, being consistent with the trends reported in the literature.

The mean reported age for PA is at the 4th decade of life, without gender predilection (8). Differently, in our study it was more common in younger patients with a slight female predominance, despite of the sample size limitation. Although previous studies have reported that the mandible is the most affected jawbone in cervicofacial cases and the maxilla in PA, this distribution is not constant in the literature (8,19). In our cases, the anterior region was more affected, particularly the lower incisors. Even though PA would be usually asymptomatic and detected in routine radiographic work-up, it may cause evident swelling, fistula or even purulent discharge and painful abscess (20), as observed in two of our cases. Previous studies have found no specific correlation of PA with inadequate root filling during endodontic treatment, even though long-lasting and refractory periapical lesions may be observed after apparently well-performed endodontic treatment in some cases (8,16). Together, these findings can suggest that radiolucent periapical lesions that are detected in unusual locations such as the anterior mandible (17) and disclose common clinical features associated with PA may be helpful to suspect of this condition.

Radiologically, PA usually presents as well-defined radiolucent lesions located in the apical region of maxillary or mandibular teeth (8). In our cases, all PA were radiolucent and half of them showed well-defined borders. Although it is more likely associated with an infected/necrotic or endodontically treated tooth (21), we, additionally, report one case of a residual cyst displaying a large amount of actinomycotic colonies, which reinforce the theory that these microorganisms can grow as extraradicular biofilms independently of an active intraradicular source of infection (22). Notwithstanding, this radiological appearance seems to be not specific for PA characterization since conventional apical periodontitis such as periapical cysts and granulomas can show similar presentation (17).

Several technics, including anaerobic bacterial culture, biochemical tests, gas chromatography, immunohistochemistry, as well as genetic and molecular tests, have been reported to assess the actinomycotic nature of cervicofacial and periapical infections (8,19-23). However, some limitations of these methods include complexity, cost burden, accessibility, and the lack of use in routine daily practice. Our results reinforce the usefulness of conventional HE-stain and additional histochemical stains such as Brown & Brenn and Grocott, which could be sufficient for establishing the diagnosis of PA when combined with clinical and radiological information. It is worth to mention that nocardiosis can also show very similar histopathological features, although periapical cases are extremely rare and the infection is almost restricted to immunocompromised patients, unlike PA (24). An additional caution must be used in interpreting Grocott stain to avoid misdiagnosis such as eumycetomas which are true fungal infections that are negative for Gram’s staining (25).

The composition of actinomycotic colonies remains also controversial. Even though it was originally believed to consist in true sulfuric granules, some authors have contradicted this suggestion (5,11). The current EDX findings confirmed that the amorphous material observed in PA lesions is sulfur- and calcium-rich as formerly believed. Some studies have elucidated that these colonies are part of a defense mechanism of Actinomyces to escape the immune system vigilance, which has been suggested to play a key role in the capacity of these organisms to grow as complex and resistant extraradicular biofilms (11,21).

The treatment of PA is mostly based on small case series or single case reports. Root filling or endodontic retreatment is usually recommended for elimination of the intraradicular source of infection, with or without perirradicular surgery when an extraradicular infection is believed to sustain a persistent periapical lesion, as well as dental extraction for elimination of the local infection. Antibiotic therapy, for this reason, may be of limited value, regarding evidences that it cannot reach the apical necrotic canal at adequate concentrations (26). Although there is no consensus on the type of antibiotic, penicillin may be the first choice in symptomatic and acute cases, where it may show any result (26). All cases included in the present study, due to its methodological characteristics, were managed by surgery.

In conclusion, our findings suggest that PA is an uncommon condition that is heterogeneous and nonspecific, both clinically and radiologically. Conventional HE-stain in addition to histochemical stains can be helpful to achieve the diagnosis of PA. Ultrastructural findings may explain the ability of these microorganisms to escape the immune system and the Actinomyces capacity to grow as extraradicular biofilms, even without an active primary dental root infection. Clinicians and surgeons should submit any surgically removed periapical tissue for histopathological analysis in order to reach a clear diagnosis and gain a better understanding of the role of microorganisms such as Actinomyces in periapical infections.
